# Human papillomavirus genotypes associated with cervical precancerous lesions and cancer in the highest area of cervical cancer mortality, Longnan, China

**DOI:** 10.1186/s13027-017-0116-y

**Published:** 2017-01-25

**Authors:** Jin Zhao, Zhong Guo, Qiang Wang, Tianbin Si, Shuyan Pei, Chenjing Wang, Hongmei Qu, Jianbin Zhong, Ying Ma, Cong Nie, Dan Zhang

**Affiliations:** 1 Medical College of Northwest University for Nationalities, Lanzhou, 730030 People’s Republic of China; 2 No.1 Hospital of Longnan City, Longnan, 746000 People’s Republic of China; 3 Gansu Provincial Cancer Hospital, Lanzhou, 730050 People’s Republic of China

**Keywords:** Human papillomaviruses, Cervicitis, Cervical intraepithelial neoplasia grade I to III, Invasive squamous cell carcinoma

## Abstract

**Background:**

The mortality of cervical cancer in Longnan is as high as 39/10 million, ranking first in China.

**Methods:**

Between 2012 to 2016, 329 samples with cervicitis, cervical intraepithelial neoplasia grade 1 to 3 (CINI to III), and invasive squamous cell carcinoma (SCC) were collected. HPV genotypes were examined with a validated kit for 23 different HPV subtypes.

**Results:**

Compared to cervicitis, the HPV positivity is significantly higher in CINI, CIN II/III, and SCC (38.60%, 74.60%, 87.50% and 89.05%, *P <* 0.001) and the positivity is also higher in SCC compared to CINI (*P <* 0.01). The most frequently detected genotypes were HPV16 in cervicitis, HPV16, 58 and 52 in CINI and CIN II/III, and HPV16, 58 and 18 in SCC groups. HPV16 positivity in cervicitis, CINI, CIN II/III, and SCC patients were 45.46%, 46.81%, 60.32% and 78.69%, respectively. Compared to cervicitis and CINI, the odds ratios (OR) for SCC in HPV16 positive patients were 2.96 (95% confidence interval [CI]: 1.09–8.00, *P <* 0.05) and 4.20 (95% confidence interval [CI]: 2.05–8.61, *P <* 0.001), respectively. In addition, the multiple infections in cervicitis, CINI, CINII/III and SCC group are 9.09%, 27.66%, 26.98% and 25.41% and HPV16 + 58 was the most common combinations.

**Conclusion:**

These findings highlight the key role of HPV16, 58, 52 and 18 in the development of CIN and SCC in Longnan women and a fully aware of regional differences in HPV genotype distribution are tasks for cervical cancer control and prevention.

## Background

Human papillomaviruses (HPV), double-stranded and non-enveloped DNA viruses (7 ~ 8 kb long), are a group of remarkably diverse DNA viruses from the Papillomaviridae family, which are causally involved in the etiology of various benign and malignant neoplastic lesions of mucosal and skin epithelium [1, 2]. Currently, more than 200 different HPV genotypes have been identified. Genotypes HPV16, 18, 31, 33, 35, 39, 45, 51, 52, 56, 58 and 59 are regarded as high risk types (hr-HPV) because they are identified in high-grade squamous intraepithelial lesions (HSIL) and invasive cervical cancer tissues [3–5]. On the other hand, the genotypes HPV6 and 11 are considered as low-risk types [4, 6].

Cervical cancer (CC) is a major fatal malignancy among women, causing about 265,700 deaths annually world-wide. Nearly 90% of cervical cancer deaths occur in developing countries, such as China [7]. The cervical cancer incidence in China is high, with 132,300 new cases each year, yielding a rate of 27 per 100,000 women [8]. Epidemiological studies and experimental data verify that persistent HPV infection is considered to play a key role in the development of CC [9]. Cervical intraepithelial neoplasia (CIN) reflects a continuous and progressive CC process, and high grade squamous intraepithelial lesions (HSIL) with HPV infection, can develop and progress to CC over a period of 8 to 12 years [10].

The prevalence of HPV infection and the reported type-specific distribution varies greatly by geographic region and ethnicity. For example, HPV16 is slightly more prevalent in Europe and North America, HPV 31 is more prevalent in South/Central America, HPV 33 and 45 are more prevalent in Africa, and HPV 52 and 58 are more prevalent in Asia [9, 11–17]. Furthermore, the data from mainland China indicated that HPV16, 18, 33, and 58 were the most common types in women with CC in Henan, central China [18], whereas HPV16, 58, 18 and 33 were the most prevalent types in CIN2+ (high-grade cervical lesions, including CINII/III, and CC) in women in Liaoning, northeast China [19], and HPV16 and 58 were the most common types in CC and high-grade precancerous lesions in Chengdu, southwestern China [20].

Longnan of Gansu Province, located in the remote areas of Northwest of China, is the high incidence areas of cervical cancer and cervical cancer mortality as high as 39/10 million, ranking first in China [21]. This study aims to investigate the prevalence and distribution of HPV oncogenic genotypes in patients with cervicitis, CINI, CINII/III, or invasive squamous cell carcinoma (SCC) in Longnan. The results will help to establish more cost-effective follow up and guiding significance for cervical cancer prevention.

## Methods

### Study subjects

A total of 329 Longnan patients aged 17 ~ 79 years samples were initially included in the present study between January 1, 2012, and January 30, 2016: 57 in the cervicitis group, 63 in the CINI group, 72 in the CINII/III group, and 137 in the SCC group. All the samples were obtained from patients who underwent biopsies with colposcopy or advanced operations. In all of samples, 305 were obtained at the No.1 Hospital of Longnan City as well as 24 samples from Gansu Provincial Cancer Hospital. All patients gave written informed consent for their participation. This study has been approved by the Ethics Committees of Northwest University for Nationalities prior to its start. All the samples were formalin-fixed and paraffin-embedded. All specimens were evaluated by at least 2 experienced pathologists in their respective hospital’s pathology department. Cervicitis, CINI, CINII/III and squamous cervical cancer (SCC) were diagnosed according to the standard criteria [22].

### HPV genotype screening using the human papillomavirus genotyping kit

The Human Papillomavirus Genotyping kit for 23 Types was produced by Yaneng Bioscience co., LTD (Shen Zhen, China), and the kit was applied for with permit number 3400994 in 2008 by the Fresh Armed State Drug Administration, China. The kit was used to perform Polymerase chain reaction (PCR) to amplify the L1 gene in conjunction with reverse dot blot (RDB) analysis to identify the HPV subtypes. This method offers a simple testing strategy involving a membrane chip that can detect infections from multiple HPV subtypes, including 18 high-risk types (HPV16, 18, 31, 33, 35, 39, 45, 51, 52, 53, 56, 58, 59, 66, 68, 73, 83 and MM4) and 5 low-risk types (HPV 6, 11, 42, 43 and 44). This method had a sensitivity of 103 copies/ml and a specificity of 99%; β-globin was used as an internal positive control [23].

### DNA extraction and polymerase chain reaction (PCR) conditions

DNA was extracted from 4–5 serial sections (4 μm thick) by hot dehiscing using the Human Papillomavirus Genotyping kit for 23 Types, according to the manufacturer’s instructions. The tissues prepared for extraction included representative tumor tissues and the adjacent normal tissue. The quality of the extracted DNA was verified using a spectrophotometer (260/280 nm ultraviolet light). The extracted DNA was concentrated by high-speed centrifugation at 4 °C. For each PCR reaction, 5 μL extracted concentrated DNA was used in a final reaction volume of 25 μL. The PCR amplification conditions were as follows: preheating at 95 °C for 10 min, followed by 40 cycles of denaturation at 94 °C for 30 s, annealing at 42 °C for 90 s, and extension at 72 °C for 30 s, with a final extension at 72 °C for 5 min. The amplification were then denatured and subjected to hybridization.

To test the quality of the DNA, the kit was also used to amplify the housekeeping gene β-globin within the same reactions as an internal positive control. We also used a verified HPV multiple infection cervical intraepithelial neoplasia sample as a positive control and distilled water as a negative control; all control samples were subject to the same treatments and processed at the same time as the experimental samples. To ensure the samples were not contaminated within the lab, every test was carried out with fresh wash buffer and all samples were independently tested in two isolated labs by blind assignment.

### HPV detection and typing

We use the reverse dot blot (RDB) method for HPV detection and typing. The 25 μL reaction volumes containing the amplified fragments were hybridized to the dot blot membrane in 6 mL hybridization solution (2 × SSC, 0.1%SDS) at 51 °C for 2 h. After a stringent wash, the hybridized membrane was probed by adding a streptavidin-horseradish peroxidase conjugate (which binds to the biotinylated PCR products) and a substrate (3,3’,5,5’-Tetramethylbenzidine) to generate a blue precipitate at the site of the probe dot. The results were inspected by macroscopic observation, and the results were deemed reliable when the PC (positive control) dot appeared as a clear round blue dot. A clear round blue dot was scored as positive for the corresponding HPV subtype, a dilute blue dot was scored as weakly positive, and the absence of a dot was scored as negative.

### Statistical analysis

Statistical analysis was performed using SPSS version 19.0 (SPSS, Chicago, IL). Differences between groups were examined using the *χ* ^2^, or Fisher’s exact probability test according to the characteristics of the data distribution. The odds ratio (OR) and relative 95% confidence interval (CI) were calculated. The significance level α was set at 0.05.

## Results

### Genotypes detected in Cervicitis, CINI, CINII∕III and SCC

**Fig. 1:**
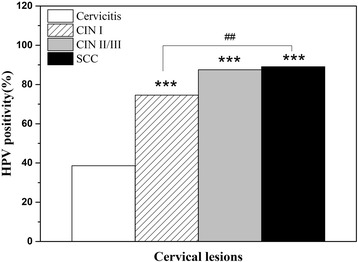
Prevalence of HPV infection in the study groups. *** *P* < 0.001 CINI, CINII/III, SCC vs. cervicitis; ## *P* < 0.01 SCC vs. CINI. Percentages for co-infections with two or more HPV strains were calculated separately for each one. Abbreviations: HPV, human papillomavirus; CIN, cervical intraepithelial neoplasia; SCC, squamous cell carcinoma

We identified 329 eligible patients with a mean age of 43.5 years (range, 17 ~ 79) from the medical records. Of these patients, 57 were confirmed as cervicitis, 63 were CINI, 72 were CINII∕III, and 137 cases were SCC. The HPV positive rates in the cervicitis, CINI, CINII∕III and SCC were 38.60% (22/57), 74.60% (47/63), 87.50% (63/72) and 89.05% (122/137), respectively; the HPV positive rate was significantly higher in CINI, CINII∕III and SCC than that in cervicitis (*P <* 0.001). and the positivity is also higher in SCC compared to CINI (*P <* 0.01. Fig. 1).

**Fig. 2:**
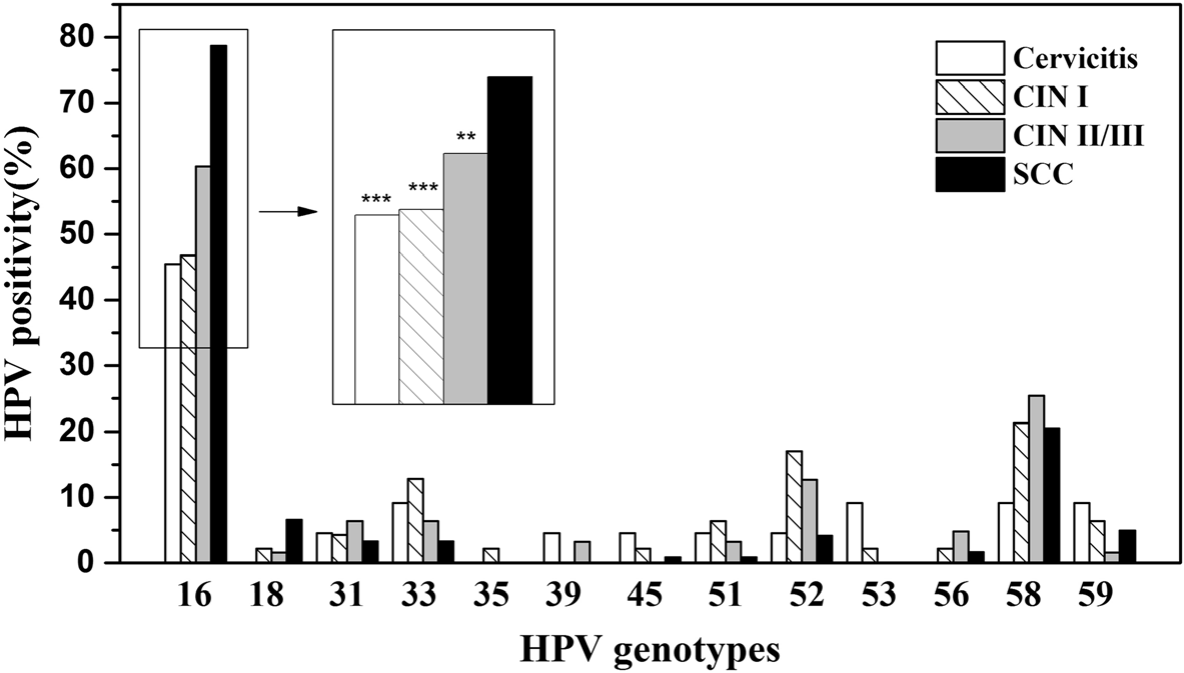
Distribution of HPV infection in the study groups. ** *P* < 0.01 vs. SCC; *** *P* < 0.001 vs. SCC. Percentages for co-infections with two or more HPV strains were calculated separately for each one. Abbreviations: HPV, human papillomavirus; CIN, cervical intraepithelial neoplasia; SCC, squamous cell carcinoma

The distribution of HPV genotypes according to cervical lesions is shown in Fig 2. HPV16 was the most common genotype in cervicitis, accounting for 45.46% (10/22). In the CINI group, HPV16 was the most common genotype, accounting for 46.81% (22/47), followed by HPV58 (21.28%, 10/47), HPV52 (17.02%, 8/47), HPV33 (12.77%, 6/47), HPV 51 (6.38%, 3/47) and 59 (6.38%, 3/47). For the CIN II∕III group, HPV16 was also the most common genotype, accounting for 60.32% (38/63), followed by HPV58 (25.40%, 16/63), HPV52 (12.70%, 8/63), HPV31 (6.35%, 4/63) and 33 (6.35%, 4/63). The 5 most common genotypes in patients with SCC were HPV16 (78.69%, 96/122), HPV58 (20.49%, 25/122), HPV18 (6.56%, 8/122), HPV59 (4.92%, 6/122) and 52 (4.10%, 5/122), in descending order. None genotype of the differences between groups were significant except HPV16 infection in SCC compared to cervicitis, CINI and CINII∕III (*P <* 0.001, *P <* 0.01. Fig. 2).

### OR for HPV16 infection

Because HPV16 was significantly higher in invasive SCC, logistic regression analysis was used to calculate the odds ratios (OR) and 95% confident intervals (95%CI) of such infections. Compared to cervicitis and CINI, the odds ratios (OR) for SCC in HPV16 positive patients were 2.96 (95% confidence interval [CI]: 1.09–8.00, *P <* 0.05) and 4.20 (95% confidence interval [CI]: 2.05–8.61, *P <* 0.001, respectively (Table 1).

**Table 1:** ORs of HPV16 for SCC

OR	95% CI	P
2.96 (Compared to cervicitis)	1.09 – 8.00	< 0.05
4.20 (Compared to CIN I)	2.05 – 8.61	< 0.001

*Abbreviations*: *HPV* human papillomavirus, *CIN* cervical intraepithelial neoplasia, *SCC* squamous cell carcinoma, *OR* odds ratio, *CI* confidence interval

### HPV genotypes of multiple infections in Cervicitis, CINI, CINII∕III and SCC

Some women were infected with 2 or 3 types of HPV simultaneously (Table 2). Compared to cervicitis (9.09%, 2/22), more patients with CINI, CIN II∕III and SCC had multiple HPV infections, however, as the cervical lesion grade increased, the prevalence of multiple HPV infections gradually deceased (27.66%, 13/47; 26.98%, 17/63 and 25.41%, 31/122, respectively). Double infections accounted for the majority of multiple infections, 4.55%(1/22), 21.28%(10/47), 20.64%(13/63) and 21.31%(26/122) for cervicitis, CINI, CIN II∕III and SCC respectively. Of those, HPV16 + 58 was the most common combinations, and HPV52 + 33 in CINI subgroup, HPV16 + 33 and HPV16 + 52 in CIN II∕III subgroup were also common genotypes combination. In the cervicitis, CINI, CIN II∕III and SCC subgroups, 4.5%(1/22), 6.38%(3/47), 6.35%(4/63), 4.10% (5/122) of patients had triple HPV infections. The combinations and prevalence of the HPV genotypes in multiple infections for each cervical lesion group and subgroup are specified in Table 3.

**Table 2:** Simple and multiple of HPV infections in the study groups

Genotype	Simple infections	Multiple infections		
		Double	Triple	Total
cervicitis	90.91% (20/22)	4.55% (1/22)	4.5% (1/22)	9.09% (2/22)
CIN I	72.34% (34/47)	21.28% (10/47)	6.38% (3/47)	27.66% (13/47)
CIN II/III	73.02% (46/63)	20.64% (13/63)	6.35% (4/63)	26.98% (17/63)
SCC	74.59% (91/122)	21.31% (26/122)	4.10% (5/122)	25.41% (31/122)

*Abbreviations*: *HPV* human papillomavirus, *CIN* cervical intraepithelial neoplasia, *SCC* squamous cell carcinoma

**Table 3:** Combinations of HPV types in multiple infection in the study groups

Genotype	Cervicitis	CIN I	CIN II/III	SCC
Conbinations of HPV types in double infections				
HPV16+18				1.64% (2/122)
HPV16+33			4.76% (3/63)	1.64% (2/122)
HPV16+52			4.76% (3/63)	
HPV16+58		4.26% (2/47)	6.35% (4/63)	9.84% (12/122)
HPV16+other types		6.38% (3/47)	1.59% (1/63)	5.74% (7/122)
HPV51+11		2.13% (1/47)		
HPV52+31		2.13% (1/47)		
HPV52+56				0.82% (1/122)
HPV52+33		4.26% (2/47)		
HPV58+53	4.5% (1/22)			
HPV58+56			1.59% (1/63)	
HPV58+59				0.82% (1/122)
HPV58+other types			1.59% (1/63)	0.82% (1/122)
Combinations of HPV types in triple infections				
HPV16+18+33				0.82% (1/122)
HPV16+18+59				0.82% (1/122)
HPV16+31+33			1.59% (1/63)	
HPV16+56+81				0.82% (1/122)
HPV16+58+31				0.82% (1/122)
HPV16+58+33		2.13% (1/47)		
HPV16+58+43			1.59% (1/63)	
HPV16+59+43				0.82% (1/122)
HPV18+33+35		2.13% (1/47)		
HPV18+58+31			1.59% (1/63)	
HPV45+53+59	4.5% (1/22)			
HPV58+31+42			1.59% (1/63)	

*Abbreviations*: *HPV* human papillomavirus, *CIN* cervical intraepithelial neoplasia, *SCC* squamous cell carcinoma

## Discussion

Our data showed that 38.60% of patients with cervicitis, 74.60% with CINI, 87.50% with CINII∕III, and 89.05% with SCC were positive for HPV DNA. HPV infection rates were significantly higher in CINI, CINII∕III, and invasive SCC than in cervicitis patients, but they never reached 100%. In addition, in our population, HPV 16 was the dominant genotype and HPV 33, 53, 58 and 59 were the second dominant genotypes for cervicitis. HPV genotypes 16, 58, and 52 were the dominant high-risk HPV in patients with CINI and CINII∕III, however for invasive SCC, the dominant high-risk HPV were 16, 58 and 18. Besides of the genotypes suggested above, HPV 6, 11, 31, 33, 51, 56 and 59 also were detected. In some reports in which HPV16, 18, and 45, HPV16, 18 and 33 or 16, 18 and 58 were most commonly detected in cervicitis [11, 24–26], CINI, CINII∕III and SCC patients. Despite the small sample size of our subgroup, the dominant genotypes remained stable across the cervicitis, CINI, CINII∕III and SCC groups, which supported the credibility of our data. In addition, HPV16 was significantly associated with SCC, the ORs were 2.96 (CI: 1.09–8.00) and 4.20 (CI: 2.05–8.61, respectively, when compared to cervicitis and CINI. The absence of a similar significant association between other genotypes may have been due to the small size of study groups.

In our group of CINI, CINII∕III and SCC patients, the positivity for HPV DNA are consistent with those in most other studies [11, 24–30]. For example, the reports from Shanghai of 239 patients with CINI and USA of 411 patients with CINII∕III, in which the rates of HPV DNA positivity were 74.9% and 82.0–92.0%. For the regions of worldwide, the rate of HPV DNA positivity in SCC was 87.0–90.9%. In addition, the positivity of HPV16 infection in CINI, CINII∕III and SCC patients is 46.81%, 60.32% and 78.69% respectively, which is also consistent with the most reports [11, 24, 31, 32]. These evidences further suggested that the performance of HPV detection kit is credible and our data likely reflect the real association pattern between HPV infection and cervical cancer or precancerous lesions in local area.

The distribution of dominant HPV genotypes showed obvious regional differences [33]. HPV18 is reported to be one of the two most carcinogenic HPV genotypes (HPV16 and 18), accounting for 10 ~ 15% of cervical cancers [11, 24, 32]. In our population, the prevalence of HPV18 in patients with cervicitis, CIN I, CIN II/III and invasive SCC were 0%, 2.13%, 1.59% and 6.56%, respectively. The overall prevalence rate of HPV18 is very lower, but consistent with a recent Korea report, where only 6 (2.5%) out of the 243 high-risk HPV positive subjects showed HPV18 infection [34]. HPV52 is also considered as a high-risk genotype and is especially frequent in Northern America, Africa, and Asia [35, 36]. In our population, the prevalence of HPV52 in patients with cervicitis, CIN I, CIN II/III and invasive SCC were 4.55%, 17.02%, 12.70% and 4.10%, respectively and was largely higher than in other reports, in which HPV52 prevalent rates ranged up to 2.4% in women with normal cytological findings, 5.1% in women with CIN II/III and were 2.5% in women with SCC [36]. In present study, HPV 58 is a dominant high-risk genotype and the prevalence of HPV58 in patients with cervicitis, CIN I, CIN II/III and invasive SCC was 9.09%, 21.28%, 25.40 and 20.49%, respectively. Although the overall prevalence rate of HPV58 in our research is very higher than the reports of Japanese and world, in which HPV58 prevalence rates are 7.0% in patients with CIN II/III and 3.3% ~ 13.3% in patients with SCC [10, 37], our result is consistent with some recent Chinese reports [33, 38], where 16.4%, 20.1%, 23.5% and 31.4% patients with cervicitis, CIN I, CIN II/III and invasive SCC and 29.1% and 24.3% patients with CIN II/III and invasive SCC showed HPV58 infection.

Our data suggested that more patients with CINI, CIN II∕III and SCC had multiple HPV infections compared to cervicitis, however, the rates of multiple HPV infections in CIN I, CIN II/III and SCC patients showed a slightly decreasing trend with severity of lesions. Although this finding is partially consistent with the results of previous studies, in which Francois et al. and Meizhu Xiao et al. found that the rates of multiple hr-HPV infections in CINII, CINIII, and CC patients declined gradually [38, 39], the rates of multiple HPV infections of our survey was significantly lower than in these reports. This difference may occur because the prevalence of HPV-positive status also vary varies among geographic locations and populations. Moreover, the small size of group in our study may be one of the reason of lower positivity of multiple HPV infections. However, in a worldwide pooled analysis of 167 adenocarcinoma of the cervix patients, the multiple-infection rate was 8.9%, obviously lower than the simple infection prevalence of 91.1% [18]. Thus, our data which reflected Multiple HPV infections might be indeed be associated with the development of cervical lesions, or women with multiple HPV infections might be more susceptible to cervical carcinogenesis, or multiple HPV infections might produce conditions that confer immunological protection against persistent infection [40].

Our data indicated that the combination of HPV16 + 58 plays a dominant role in all cervical lesion groups, contrary to findings in the Jewish Israeli population and in Austrians, but in agreement with findings in women in Beijing, China [41–43]. Thus, the results of this study strongly support the key role of HPV16 and 58 in the development of CC and CIN in women in LongNan, China.

## Conclusions

Overall, as demonstrated in our study population, genotypes 16, 58, 52 and 18 are among the predominant HPV and HPV infection increased with cervical lesion, regional differences in HPV genotype distribution and the real carcinogenic HPV revealing need to be mindful in the HPV control and prevention.
